# Large hydropower projects increase stress despite compensation efforts: Evidence from the Brazilian Amazon

**DOI:** 10.1371/journal.pone.0284760

**Published:** 2023-07-14

**Authors:** Adam Mayer, Igor Cavallini Johansen, Maria Claudia Lopez, Mariluce Paes de Souza, Emilio F. Moran

**Affiliations:** 1 Center for Earth Observations and Global Change, Michigan State University, East Lansing, MI, United States of Ameica; 2 Center for Environmental Studies and Research–Nepam, State University of Campinas, Campinas, SP, Brazil; 3 Department of Community Sustainability, Michigan State University, East Lansing, MI, United States of Ameica; 4 Programa de Pós-Graduação em Administração, Universidade Federal de Rondônia, Rondônia, Brazil; Xiangtan University, CHINA

## Abstract

Large hydropower projects continue to be built in developing nations, despite their known negative impacts. Large-scale energy projects strain local infrastructure and reduce access to infrastructure for households that live near them. Here we investigate the link between large-scale hydropower projects and stress. Our results suggest that these projects create stress through two mechanisms: strains on community resources and through the process of displacement. We also ask how compensation and resettlement programs condition these relationships. Using data from the Madeira river basin in the Brazilian Amazon, we find that hydropower projects increase stress by reducing access to energy, water, sanitation and land. Compensation provided was not sufficient to moderate this effect.

## Introduction

Over the past 50 years, developing nations have turned to hydropower to provide a stable energy source for their growing populations, facilitate economic development, and improve their position geo-politically by not relying upon expensive imported energy [1]. This has been especially true in Brazil, where the central government, regardless of who has held power, has actively encouraged large hydropower projects for decades as part of its vision for economic development. Currently, Brazil is one of the most hydropower dependent nations on earth (67%) for its electricity. As of 2019, there were 158 dams in operation and another 351 in the planning phases in the Brazilian Amazon [2], and since only 50% of the Amazon’s hydropower potential is currently used, more may be coming in the years ahead [3]. The Brazilian government has often approved the construction of dams before environmental impact assessments have been completed and despite widespread opposition against them, most notably in the case of the Belo Monte dam [4–6].

Although dams provide many benefits at a national scale (e.g., affordable and stable energy, an alternative to fossil fuels and energy independence), there are numerous negative impacts that occur in communities and regions that host large-scale hydropower dams. Among ecological impacts, perhaps the most well-documented damages are increases in deforestation and the impacts on river ecology, fisheries, and biodiversity, with consequent impact on local’s livelihoods and food security [7–11]. Hydropower may have some advantages over fossil fuels (e.g., lower carbon dioxide emissions), yet it’s ecological impacts and indirect contributions to emissions cast serious doubt on its credentials as a “green” or “clean” energy source [2, 12–15], more so in the tropics where studies have shown that dams generate greenhouse gases such as methane [16, 17]. Fan et al. [18] examined 610 dams worldwide of various sizes finding that within a 50 km radius of a dam there is consistent lower economic well-being measured in terms of GDP, decreased population size, and less vegetation measured by the Normalized Difference Vegetation Index (NDVI) than before the dams were built, and these impacts are proportional to the size of the dam. There are effects on human populations and host communities near hydropower projects. The aforementioned damages to fisheries has livelihood and nutritional consequences for peoples that rely on fisheries for income and food [8]. Undoubtedly, the largest social impact of dam construction is displacement [19–21]. An estimated 80 million people were displaced in the 20^th^ century due to dams [22]. China’s Three Gorges Dam, the largest in the world, entailed the resettlement of over 1.13 million people [23]. Authoritarian governments have often argued that hydropower and associated displacement was in the national interest and offered little to no redress to communities that were forced to move or otherwise experienced negative impacts in some way [24, 25]. Following the report of the World Commission on Dams in 2000, some governments and dam builders implemented compensation programs that provide housing, cash, credit, equipment of other types of compensation to households displaced from dam projects [25, 26]. Still, the fairness of these compensation programs is intensely debated [20, 27–29].

The “energy boomtown” literature from rural sociology provides some important insights into the processes that reduce well-being and quality of life in communities that host energy projects. This literature describes how energy infrastructure projects often located in rural places far removed from metropolitan areas with limited local capacity and unable to adjust to the sudden arrival of young, mostly male workers to the region seeking employment during a resource boom [30–32]. The arrival of these workers creates several challenges for communities. For one, local infrastructure like housing, sewage systems, and garbage services may not be able to handle the sudden influx. The energy workers might be privileged in terms of access to those resources, effectively crowding out access to key resources for the host population. This, in turn, increases stress and reduces well-being in the host community.

In the context of dam construction in developing countries, the “energy boomtown” literature has been used to show how dam construction affects not only host communities but other communities nearby [33]. In particular, constrained access to local infrastructure may be a primary mechanism by which dams reduce community well-being. Living conditions in many communities that host hydropower are often not ideal [21, 34, 35]. For example, communities near Brazil’s Belo Monte dam were promised improved water and sanitation services, but these never materialized—in fact, sanitation conditions worsened during the construction of the dam [36–38]. A community resettled because of the Belo Monte dam reported that transportation was not available to get to town to access to many services like shopping and banking in the downtown area [33]. Studying China [39], found that displacement increased food insecurity and reduced income for some resettled households. Inadequate infrastructure after resettlement was also detailed by Green and Baird [40].

In this paper we extend research on hydropower impacts, compensation and well-being by asking the extent to which the “boomtown” impacts of hydropower, such as the deterioration of infrastructure such as electricity, land, water and sewage and the experience of resettlement, influences stress levels among people living near the dams. A further goal of this paper is to understand whether compensation reduces the effect of these impacts. In doing so, we build upon the small literature that evaluates subjective well-being (e.g., stress, depression and general quality of life) in the context of hydropower projects [21, 41] and health and hydropower more generally [36, 37, 42–47].

## Displacement and compensation

Households that are displaced to make way for hydropower infrastructure are often resettled as part of a compensation program, but they may not be resettled near friends and family in their new location and experience a loss of social capital [38, 48, 49], they also may be resettled in settlements that do not have the same natural capital (losing access to common pool resources such as rivers) they use to have around, or move to a location that has land of worse quality than what they have before which creates a loss in people livelihoods [50–52]. Thus, resettled households may need to learn new skills and find new livelihoods and sources of income to be able to make a living in their new location.

Compensation strategies are meant to restore the livelihoods of people who are directly affected by the construction and operation of dams [27, 53]. Compensation is a mitigation mechanism to reduce social, economic and environmental impacts during the construction of the project and its aftermath [54]. However, compensation has been poorly implemented [27]. Dam companies and governments design resettlement programs to compensate displaced populations with cash payments or new housing [29] to prevent impoverishment and to reconstruct people’s livelihoods [18]. Yu & Xu [54] used a social impact analysis framework to study compensation policies looking at different types of wealth: material, embodied, and relational. Material wealth refers to types of wealth that can be readily measured monetarily, such as land or various kinds of household assets. Embodied wealth is carried by a person, most notably skills and knowledge acquired through education and practice. Relational wealth refers to social infrastructure (e.g., social relationships, social capital) and physical infrastructure (e.g., transportation, healthcare facilities, schools) that are not directly owned by individuals but benefits them, their household, and community. In this paper we focus on material wealth and relational wealth in the form of community infrastructure.

Compensation programs have been routinely criticized as insufficient or unfair on a number of grounds. Wang et al.’s framework [54] helps to understand the dimensions of wealth that are offered and ignored by compensation programs, in particular embodied and relational. In addition, there are many households that are impacted by dams that often receive no compensation, or even considered impacted because they live downstream from the dam [29, 55]. The World Commission of Dams estimated that more than 470 million people living downstream from dams had been impacted by dam construction, but had never been compensated.

In terms of subjective well-being, understood as the way people self-evaluate their lives or experiences [56], displacement caused by development projects may lead to depression [57]. Xi & Hwang [41] report that communities displaced due to the Three Gorges Dam in China experienced a loss of social integration, which was associated with depressive symptoms. Cao & Hwang [58] extended this work by showing that a loss of social integration and community resources increased depression. On the other hand, Randell [59] finds that some households that were resettled and compensated to make way for Brazil’s Belo Monte dam experienced improved wealth and subjective well-being, but that sample was far from typical since they were landowners with substantial land and cattle for which they were amply compensated early in the process. Mayer et al. [33] using data from the Madeira river basin in the Amazon, show that social capital was reduced during and after the construction of the dams, thereby reducing well-being. More generally, psychological stress has long been identified as one of the outcomes of large-scale development projects like dams [59–61]. Thus, prior research is consistent in finding that the process of dam construction and operation creates various strains on community wealth in the form of infrastructure (e.g., land, sewage systems), strains that reduce well-being, but more research is warranted on this topic to understand how these changes might contribute to stress.

## Research questions

Following the diverse literature outlined above, we aim in this paper to evaluate the following research questions. First, following Cao & Hwang [58] as well as the boomtown literature (e.g., England & Albrecht [31]), we ask how the deterioration of already scarce access to infrastructure (e.g., electricity, land amount, water and sewage) increases stress levels. For our second research question, we investigate if compensation can moderate the impact of these losses on stress.

## Methods

### Study region

**Fig 1:**
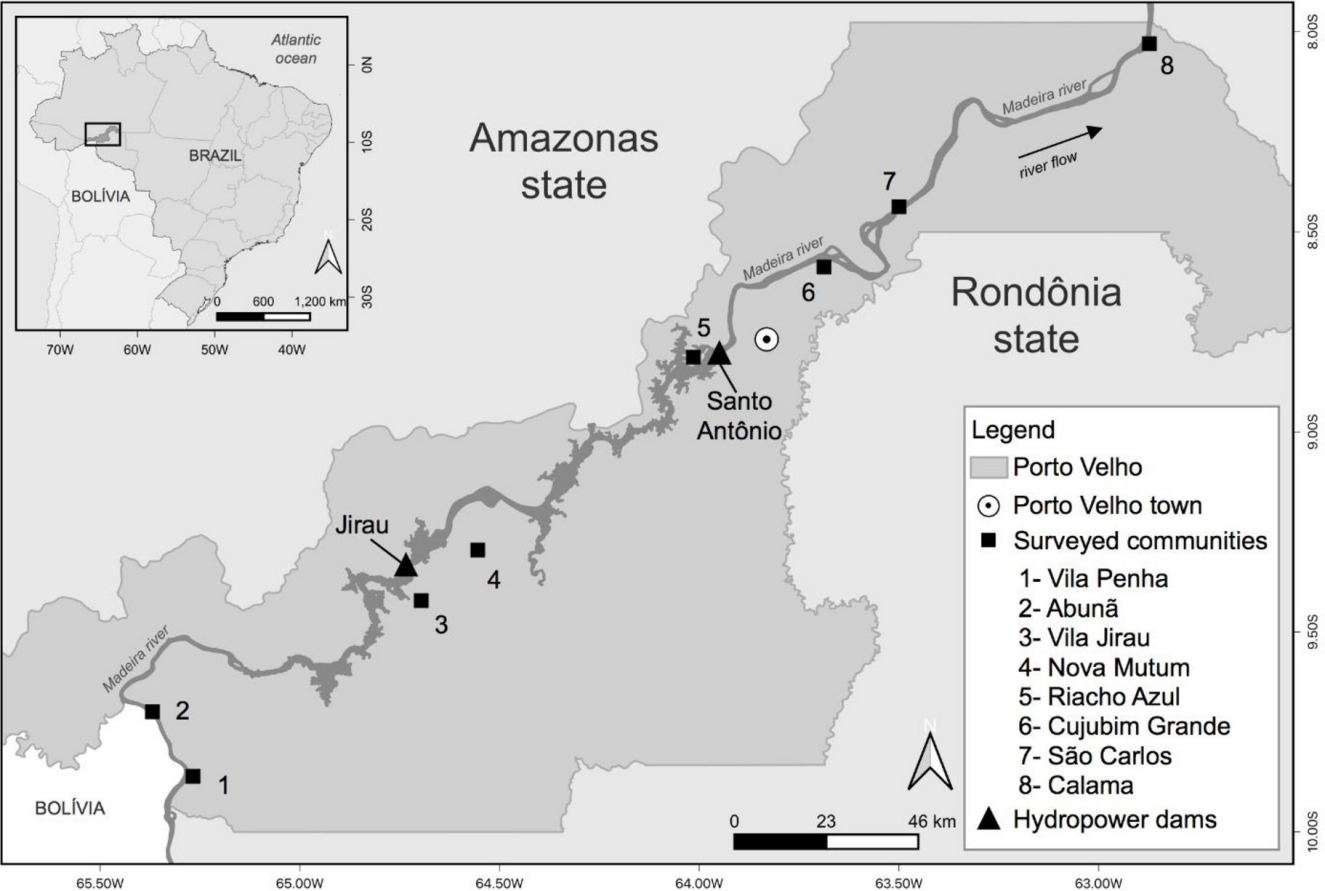
Study area: Map of the surveyed communities in the Madeira river basin, Brazil. Communities’ names are numbered from 1 to 8. Hydropower dams are represented with black squares. The gray shaded area shows the Rondônia capital, Porto Velho municipality, and the white circle with a dot in the center indicates the location of the municipality seat, Porto Velho town. Porto Velho municipality is part of the Rondônia state, in the frontier with Amazonas state. Figure created with QGIS software version 3.14, an open source Geographic Information System (GIS) licensed under the GNU General Public License (https://bit.ly/2BSPB2F). Publicly available shape files provided from the Brazilian Institute of Geography and Statistics (IBGE) website (https://bit.ly/34gMq0S). River shape file retrieved from the Brazilian National Water Agency (ANA) website (https://bit.ly/3Bx8kiI). All utilized geographical data are under the Creative Commons Attribution License (CC BY 4.0).

The current research is part of a broader, long-term research project into the effects of hydropower projects in multiple river basins in the Brazilian Amazon. This project involves regional partnerships with universities, extended qualitative interviews, qualitative field observations, survey research, and document analysis. For this paper, we rely upon data collected through survey research methodology in eight communities along the Madeira River near two large hydropower dams—the Santo Antônio and Jirau—that were constructed between 2008 and 2013 (Fig 1). Both dams were built with limited consultation with the proximate communities and construction began before environmental impact assessments (EIAs) had been completed [62–64]. The dams were funded by a combination of foreign and domestic investment [13]. Santo Antônio has an installed capacity of 3,568 MW and Jirau, of 3,750 MW. The Santo Antônio dam is 7km upstream from Porto Velho, the capital city of the state of Rondônia. Jirau is 125km upstream. Both are “run of the river” style dams that require limited water storage and allow water to flow at the same rate both upstream and downstream of the dam. Residents of the region engage in a variety of economic activities, ranging from fishing, agriculture, logging, small-scale mining, ranching, and various professions.

The construction of Jirau and Santo Antônio started with insufficient consultation with the impacted populations [62–65], in fact the dam consortia held only four public hearings in the region. Mayer et al. [66] show that participation by local communities was full of procedural injustices, and at the end community members were pressured to accept the construction of the dams. Years after the construction of the dams, reports from the field indicated that many households were still in litigation with the dam builders through the public prosecutor’s office to receive fair compensation. The dams have also contributed to flooding, which of course has multiple deleterious impacts for riverine and subsistence communities. Our collaborators in the region report that the dam builders and supportive government officials touted the dams as an economic boom for the region, but in general these promises have failed to materialize. For instance, the communities were promised fruit processing facilities that never became operational. Other research papers document the impacts to fisheries from these dams [7, 10, 67]. In addition to these community impacts, these factors could have also increased stress in the communities.

About 15% of the sample (described further below) reported that they received compensation and were resettled, but only 5% of those reported that they were given a choice as to how they would be compensated. Most (73%) reported receiving cash and nothing else. The rest had a varied mix of access to new fishing locations, boat motors, boats or canoes, inputs for agriculture, or even loans. Only one-percent of the resettled respondents stated that they had a choice in location where they would be resettled to.

### Data collection

We collected the survey data in the communities between August 2019 and March 2020 (data collection ended before the coronavirus pandemic shutdowns). Vila Penha and Abunã were not scheduled to be resettled. Yet at the time of the data collection, they were aware of possibly needing to be resetled due to unforeseen flooding levels generated by the construction of the dam. The flooded area was 67% larger than predicted in the pre-dam studies. Two communities—Nova Mutum and Riacho Azul—were created as resettled and compensated communities. In addition, Nova Mutum had a substantial neighborhood for dam staff remaining to operate the Jirau dam. Nova Mutum was built to resettle population from Mutum Parana, which was flooded to make way for Jirau dam. Riacho Azul also included resettled populations from across the other side of the Madeira river who would be flooded by the reservoir. Vila Jirau existed before the dam and became a haven for resettled people who were unhappy with the Nova Mutum planned community’s location. Cujubim Grande is largely composed of households that resettled from the 2014 flooding that led to their previous settlement collapsing into the river from the increased precipitation that year and the force of the water released to reduce water pressure on the dam. It is important to note that many of the initial residents of these communities—the initial people that were resettled—might have moved to other communities, and new people not resettled by the dams are now living in these houses. The remaining two communities, São Carlos and Calama, were downstream of the dams and the communities were not compensated nor resettled.

To develop the sampling frame, we used satellite imagery of each community, complemented by visits to communities to verify the existence of structures and identify houses and eliminate other structures (e.g., schools, churches). We numbered the homes and drew a random sample proportional to size in each community, using up to five contact attempts. Interviews were conducted by local university students that were supervised by two post-doctoral scholars headquartered in Porto Velho and all interviewers were local students fluent in Portuguese. Before we started the survey, we trained interviewers on data gathering procedures (e.g., use of tablets) and presented standard ethical guidelines used in data collection and management. Interviewers typically worked in pairs, using a numbered map to locate the sampled homes, we provided a list of alternative houses if they were unable to locate the residents from the initial draw.

After arriving at each home, the interviewers introduced themselves, explained their academic affiliation and the goals of the study and asked for the household’s consent to proceed with the interview. We did not use a written consent form because of the relatively low levels of education among the participants. Instead, oral consent was requested and recorded before the start of the survey.The study procedures were approved by the institutional review boards of Universidade Federal de Rondônia and Michigan State University. One interviewer asked questions from the household head, while the other took notes and assisted with potential distractions, such as small children. Typically, the male head of the household completed the survey, although many female heads of household also completed the interview. Table 1 provides the number of households sampled and number of completed interviews within each community, among other information.

**Table 1:** Communities sampled in the Madeira river basin, Brazil, from August 2019 to March 2020.

	Location	Number of structures in community	Completed Interviews
Vila Penha	Upstream	148	33
Abunã	Upstream	212	100
Vila Jirau	Upstream	240	71
Nova Mutum	Upstream	267	78
Riancho Azul	Upstream	82	52
Cujubim Grande	Downstream	220	78
São Carlos	Downstream	282	108
Calama	Downstream	440	151
Total		1,891	671

Note: Number of structures refers to the number of buildings that were visible from satellite imagery.

Interviews lasted an average of 90 minutes, and the survey instrument had some 338 questions, although few respondents answered all these questions due to skip patterns. For instance, there were special sections for respondents who fished or worked in agriculture as a primary source of income. In any given community, between 3–5% of households refused to participate. Once completed, the interview location was automatically geocoded. The team gave a card to the respondent with contact information for a local collaborator who could answer questions about the project. We collected data from 671 households, or 35% of the total, based on face-to-face contact for a 3.04% margin of error to achieve a 95% confidence level, although the estimation sample used in our models below is somewhat lower due to missing data, as typically occurs in survey research. The excluded data did not compromise the statistical power of the analysis, since the calculation of the sample already considered the possibility of a small fraction of missing cases. We used eleven survey questions in this analysis. Survey questions and original anonymized data are available in S1 Dataset.

### Outcome variable

This paper does not utilize a specific psychometric scale to assess stress levels [67, 68]. Our analysis is based on a question that allows the interviewee to subjectively present his/her level of emotional stress. Evaluations of well-being (e.g., stress, life satisfaction) routinely rely upon single indicators instead of additive scales [47, 69]. The advantage of our approach is that it allowed us to assess many other topics in our survey, as some scales for stress involve several questions. Respondents were asked the extent to which their emotional stress had improved, remained the same, or had increased due to the dams. We scored this variable such that higher categories indicate worse outcomes. Our understanding of stress does not center upon physiological processes (e.g., increased blood pressure) or behavioral changes to cope with stress (e.g., alcohol consumption, changes in appetite) [70]. Rather, we understand stress in a psychological sense as the appraisal of a situation as stressful, such as the construction of the dams and their impacts [67, 71, 72].

### Predictors: Compensation and community infrastructure

Respondents reported whether or not their household had been compensated and resettled. There was significant diversity of types of compensation, such that we use a binary indicator for receiving any type of compensation. Many respondents received housing, but others also received cash payments, fishing equipment, agricultural inputs, loans, or even boats. As previously mentioned, about 15% of the sample indicated that they had been resettled and compensated.

Respondents answered questions related to electricity access, land amount, water quality, water access, and sewage access after the construction of the dams. For instance, for the land amount variable, we asked: “Has the **amount** of land you and your household currently have remained the same, decreased, or increased (due to the building of the dam(s))?”. Other questions used a similar wording. Response categories included “Improved”, “Remained the same”, or in some cases “Do not have”, which were merged with “Remained the same”. We disaggregate these responses by whether the respondents were resettled and compensated or not.

### Control variables

Stress is likely influenced by a range of variables that are not related to access to basic infrastructure. We control for the sex (male, female) and education (no formal education, primary education, secondary education, and technical or advanced degree) of the interviewee, whether the home had a female head of household, and a two-category variable for community type (downstream and upstream).

### Modeling approach

First, we conducted a cross-tabulation of our outcome variable (stress) with each of the infrastructure predictor variables, running a chi-squared test for each of these relationships and testing the statistical significance of the different proportions, taking as reference p<0.05. Then, we applied statistical modeling. Our outcome variable “stress levels” is ordinal. To accommodate this distribution, we use ordinal logistic regression models to understand how our predictors influence stress levels. In S1 Table, we begin by estimating a baseline model (i.e., a model with no interaction term) and then an interaction model for each type of local infrastructure loss, comparing AIC and BIC statistics to determine if the interaction terms have improved model fit [73, 74].These models allow us to understand whether compensation and resettlement, as interaction terms, reduce the effect of a loss of infrastructure on increased stress. Then, we provide a “full” model that eschews the interaction terms but uses all of the predictors together to understand the combined effects of infrastructure changes (Table 3). In Figs 4 and 5, we turn to predicted probabilities and average marginal effects, respectively, to more intuitively understand the implications of our models, given the well-known problems with interpreting logistic regression coefficients [75].

## Results

**Fig 2:**
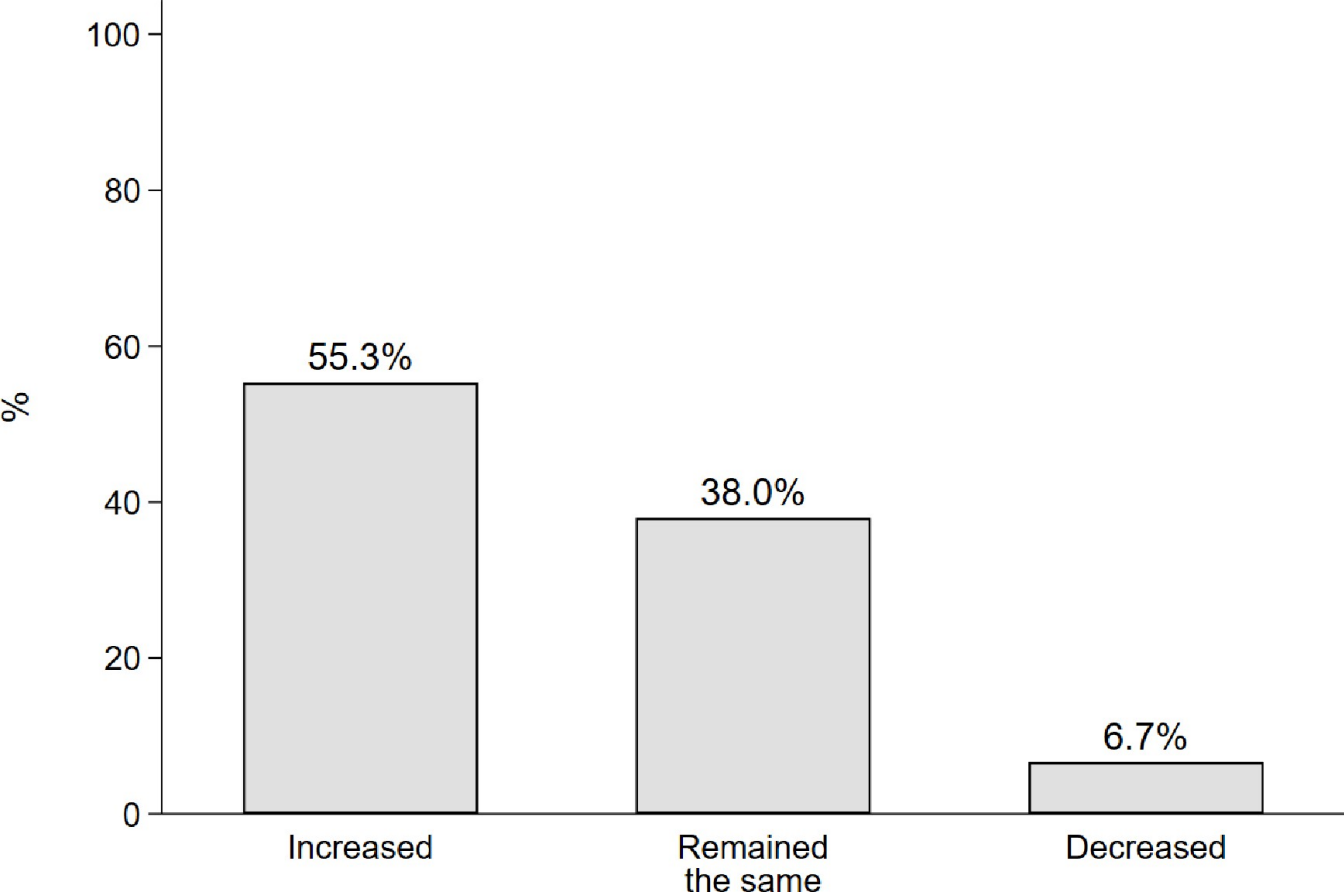
Distribution of the outcome variable “stress” based on the question. “Has the amount or level of emotional stress you or others in your household experience remained the same, decreased, or increased (due to the building of the dam(s))?”, n = 658.

As shown in Fig 2, most respondents reported that the dams had increased their stress (55.3% stated “increased”). Notably, a large minority of respondents stated “remained the same”, and relatively few indicated that the dams had decreased their stress.

**Fig 3:**
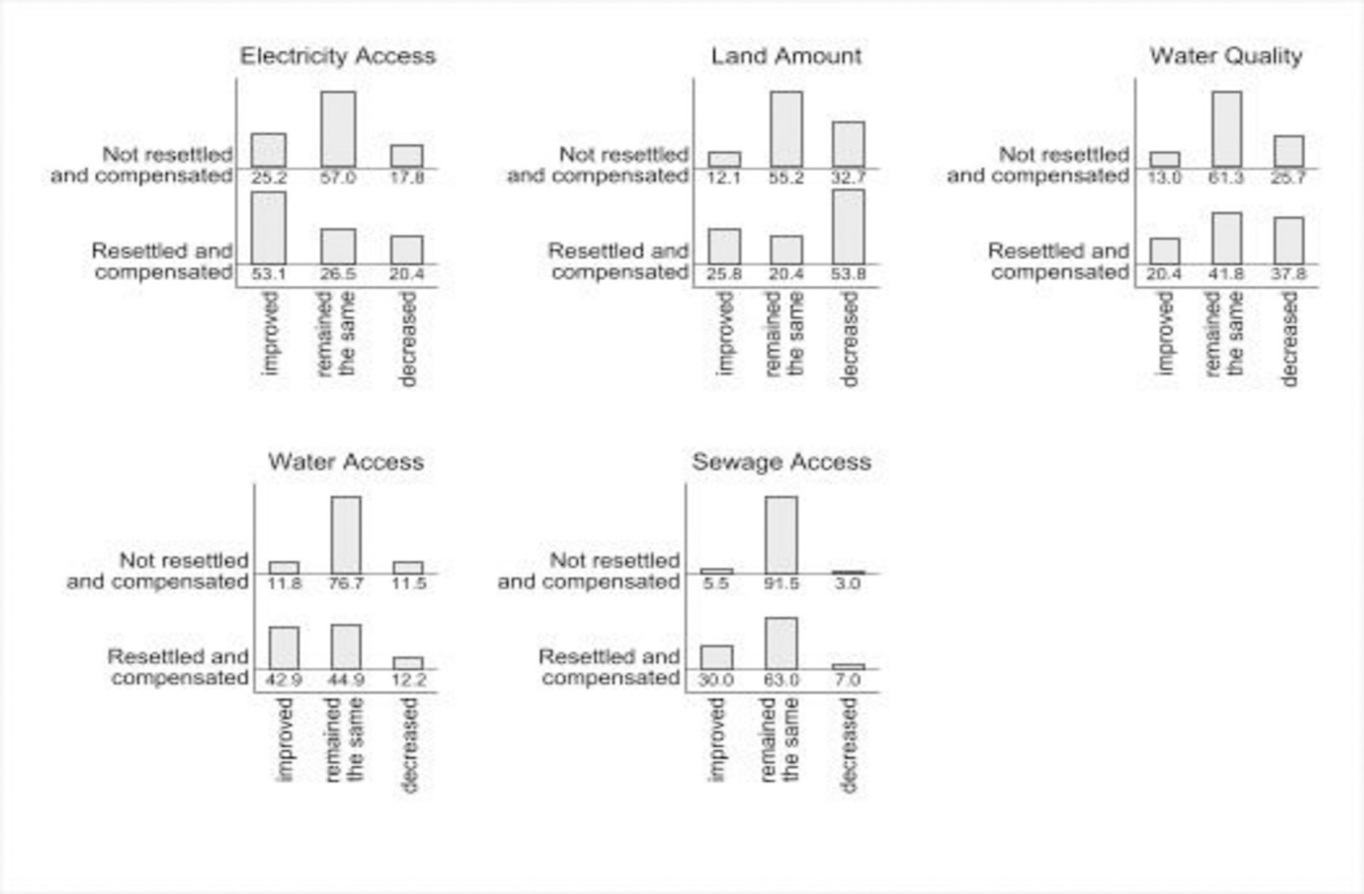
Changes in access to infrastructure by resettlement and compensation status. Percentage of respondents who were not resettled and compensated and who were resettled and compensated for each type of infrastructure loss, n = 576.

According to Fig 3, overall, respondents indicated that access to most infrastructure “decreased” or “remained the same” and the resettled and compensated group were not consistently more likely to state that their access to infrastructure had improved. Importantly, for the resettled and compensated populations, “decreased” answer reach higher proportions than in not resettled and compensated, suggesting that the new infrastructure offered to the resettled and compensated group was not sufficient.

Table 2 provides the cross-tabulation of our outcome variable (stress) with each of the infrastructure predictor variables and a chi-squared test for each of these relationships. We show the number of cases in each cell (n) and row percentages. Among those who stated that electricity access had “decreased”, 65% reported that stress had also “increased”, and among those who indicated that “land amount” had “decreased”, 66% also reported “increased” stress—in both cases the relationship between these variables is statistically significant (p<0.05). Those who reported “decreased” access to water quality or “decreased” access to water in general were also more apt to report “Increased Stress” (with 71% and 69% respectively). However, the association between sewage access and stress is not statistically significant, and this may be because many people in rural areas did not have sewage system to begin with.These descriptive results imply that a loss of infrastructure, or lack of improvements in infrastructure, is associated with increased stress due to the construction of the dams.

**Table 2:** Cross-tabulation of “stress” and community infrastructure variables of communities in the Madeira river basin, Brazil.

Stress									
	Decreased		Remained the Same		Increased				χ^2^ (p-value)
**Electricity Access**	n	%	n	%	n	%	Total	%	
Improved	20	10.526	79	41.579	91	47.895	190	100	15.611(0.004)
Remained the same	15	4.412	137	40.294	188	55.294	340	100	
Decreased	9	7.627	32	27.119	77	65.254	118	100	
**Land Amount**									
Improved	8	9.524	38	45.238	38	45.238	84	100	18.157(0.000)
Remained the same	13	4.377	126	42.424	158	53.199	297	100	
Decreased	14	6.604	58	27.358	140	66.038	212	100	
**Water Quality**									
Improved	8	8.791	37	40.659	46	50.549	91	100	28.267(0.000)
Remained the same	23	6.053	168	44.211	189	49.737	380	100	
Decreased	12	6.704	39	21.788	128	71.508	179	100	
**Water Access**									
Improved	13	12.150	38	35.514	56	52.34	107	100	12.568(0.014)
Remained the same	29	6.131	190	40.169	254	53.70	473	100	
Decreased	2	2.667	21	28.000	52	69.33	75	100	
**Sewage Access**									
Improved	7	11.667	26	43.333	27	45.000	60	100	5.345(0.254)
Remained the same	35	6.076	218	37.847	323	56.076	576	100	
Decreased	2	9.091	6	27.273	14	63.636	22	100	

S1 Table provides baseline and interaction models for each type of strain on local infrastructure, along with AIC and BIC statistics. Our baseline model for “Electricity Access” indicates that those who reported that access had “remained the same” or “decreased” experienced more stress (p<0.05). In the next model, we interact compensation and resettlement with changes in electricity access. The interaction terms are not statistically significant, and the AIC and BIC have both increased, implying that the interaction has not improved model fit—this implies that compensation did not alter the effect of decreased energy access on stress. The next two models use land amount as the primary predictor, wherein those who report that the amount of land they own has “decreased” report greater stress (p<0.001). Here again, the interaction terms are not statistically significant, and the model fit statistics imply that interaction model has a worse fit than the model without the interaction term. The lack of model fit improvement and null interaction imply that the compensation program did not reduce the effect of lost land on stress.

Turning to water quality, those who stated that water quality had “decreased”, compared to those that stated that it had “increased”, have higher stress levels (p<0.01). Once again, the interaction models show that compensation and resettlement did not buffer the effect of a loss of water quality on stress levels, as the interaction terms are not statistically significant, and the AIC and BIC statistics are larger in the interaction model. For water access, the result is similar, i.e., “decreased” water access is associated with higher stress levels (p<0.05) and the interaction with resettled and compensated did not improve the model fit. Our final models for sewage access indicate that individuals who report that sewage access “remained the same” are more likely to state that stress had “increased” (p<0.05). But, as with all our other models, the interaction terms are not statistically significant, and the model fit statistics imply a lack of moderation. That is, in all our models, we are finding that the stress-increased effect of changes in infrastructure is not altered by compensation. Respondents were comparing infrastructure conditions before and after the dams and reported increased stress, but compensation did not reduce the effect of a loss of infrastructure on stress. Our direct effect for community type was significant in most models, with upstream residents reporting increased stress. We also note that our variable for resettlement and compensation has a statistically null effect in all the models. That is, compensation does not seem to reduce stress directly.

**Fig 4:**
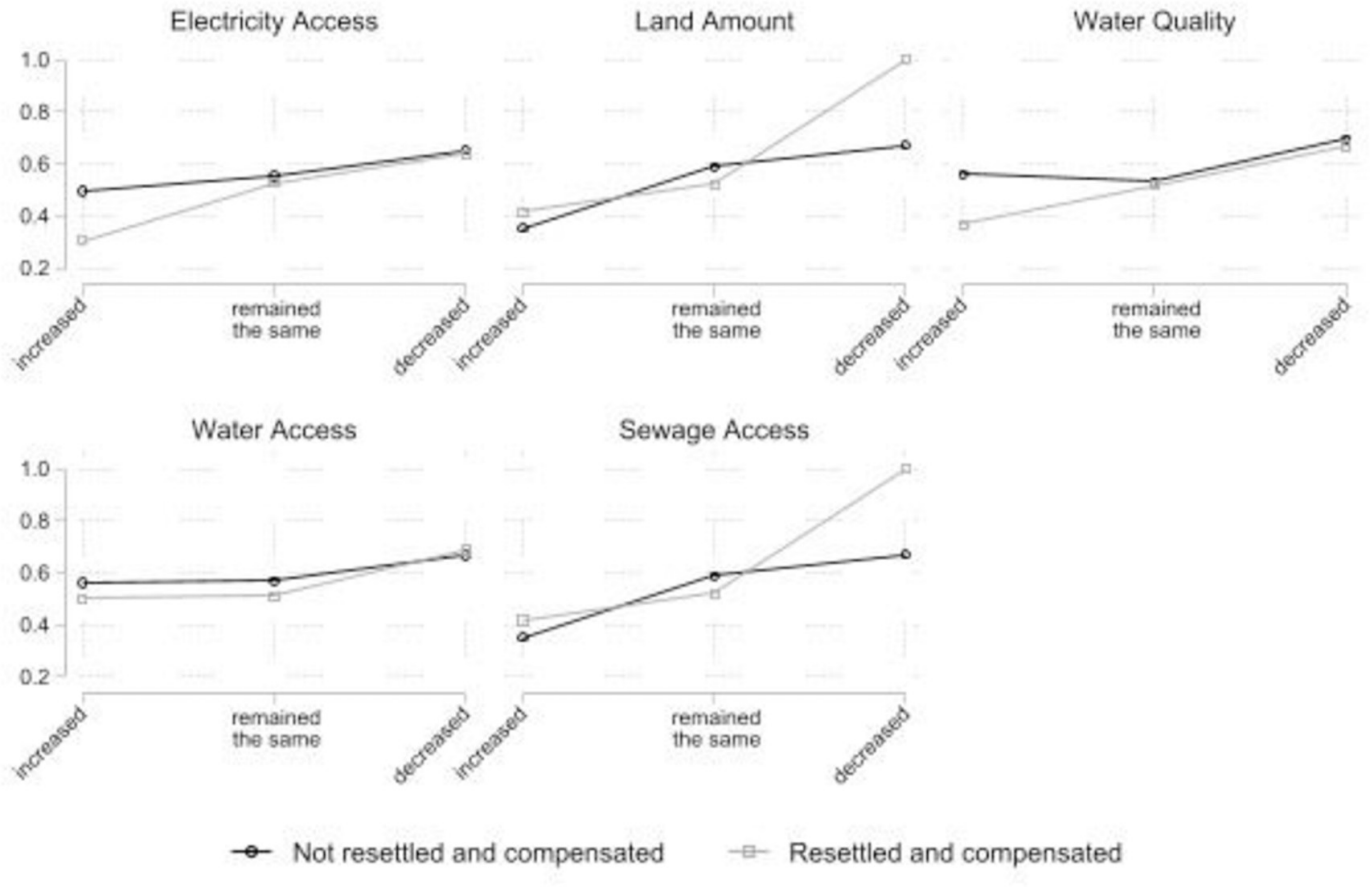
Probability of “increased stress” by interaction of the infrastructure variables with resettlement and compensation. Estimates were derived from the “Interaction” ordinal logistic regression models reported in S1 Table with all other predictors held at their observed values.

As we noted above, ordinal logistic regression coefficients are difficult to directly interpret, and it is considered a best practice to not rely purely upon p-values to determine if an interaction is important [75]. Fig 4 displays the interaction between the loss of infrastructure and resettlement and compensation, expressed as the probability of “increased” stress. The graphic corroborates the largely null findings for the interactions reported in S1 Table—with the possible exceptions of the “land amount” and “sewage access”. Further, the gap between the compensated and resettled curve and the curve for those that were not resettled and compensated is miniscule, again implying that compensation likely did not redress the stress induced by a loss of infrastructure.

Next, in Table 3 we turn to our “full” model that drops the interaction terms and uses each loss of service as predictors. We find that, compared to those who reported that electricity access improved, those who stated that it “decreased” were more likely to state that their stress had “increased”. We find similar results for land amount, but the effect of water quality and water access is not statistically significant in this model, implying that the effect of a loss of water quality and access is less robust.

**Table 3:** Regression model for stress with all infrastructure predictors of communities in the Madeira river basin, Brazil.

	b(se)
**Electricity Access** (*ref*. *improved*)	
Remained the Same	0.455*
	(0.21)
Decreased	0.717*
	(0.29)
**Land Amount** (*ref*. *improved*)	
Remained the Same	0.425
	(0.26)
Decreased	0.885**
	(0.27)
**Water Quality** (*ref*. *improved*)	
Remained the Same	-0.111
	(0.30)
Decreased	0.614
	(0.33)
**Water Access** (*ref*. *improved*)	
Remained the Same	-0.332
	(0.32)
Decreased	-0.293
	(0.41)
**Sewage Access** (ref. improved)	
Remained the Same	0.809*
	(0.38)
Decreased	1.164
	(0.69)
**Status** (*ref*. *not resettled and compeansated*)	
Resettled and Compensated	-.054
	(0.279)
**Sex (*ref*. *female*)**	
Male	-0.072
	(0.23)
**Education** (*ref*. *no formal education*)	
Primary Education	0.372
	(0.27)
Secondary	0.648*
	(0.31)
Post-Secondary/ Technical	0.601
	(0.40)
**Sex of the head of household** (*ref*. *male*)	
Female head of household	0.109
	(0.25)
**Community type** (*ref*. *downstream*)	
Upstream	0.312
	(0.20)
AIC	915.342
BIC	1001.612

Note: N = 554. *** for p<0.001

** for p<0.01 and * for p <0.05. Data was gathered in 2020 in the Madeira River Region of the Amazon Basin.

**Fig 5:**
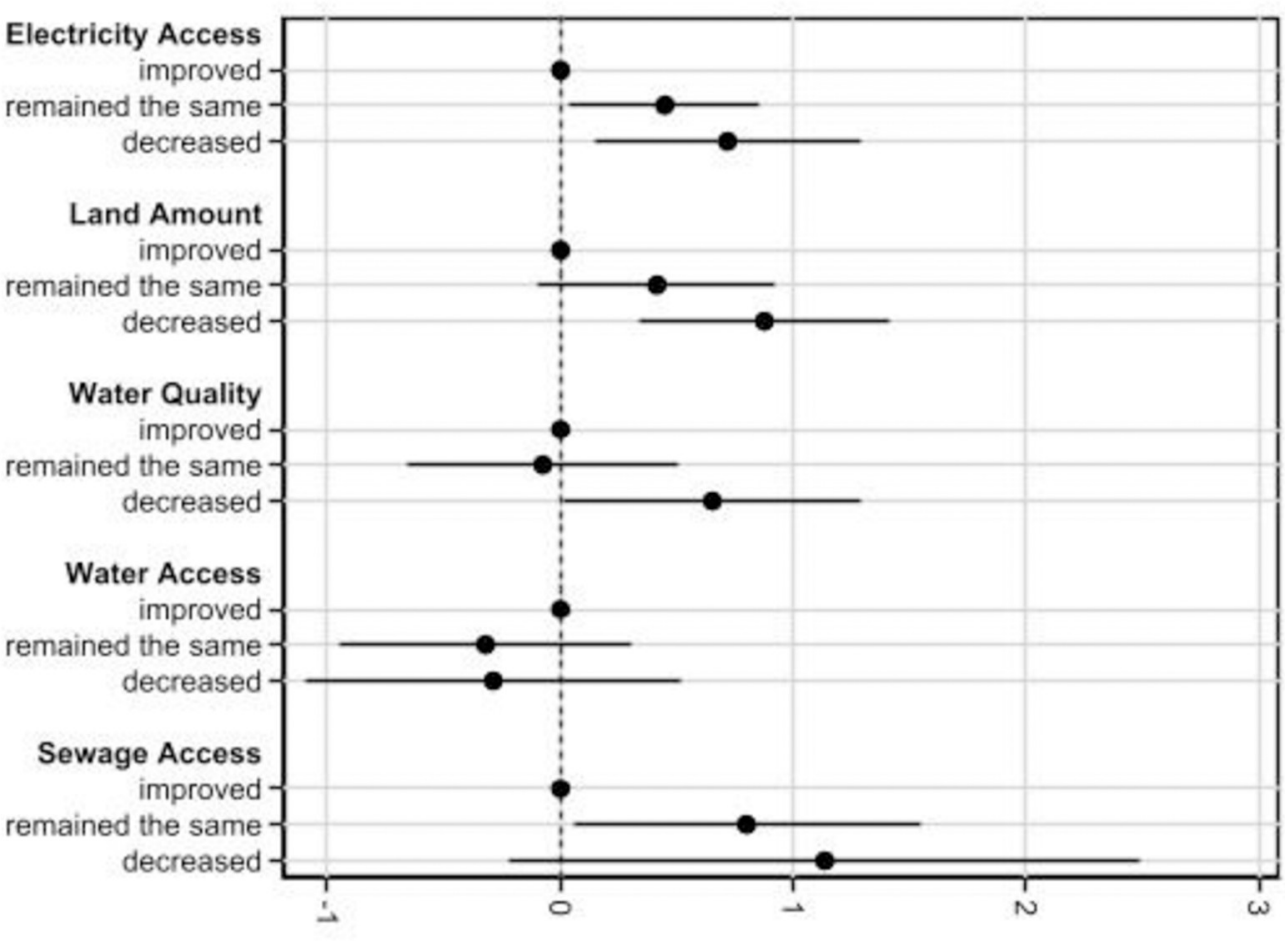
Average marginal effects with 95% confidence intervals derived from the “Full” model (Table 3). The AMEs show the change in probability of “increased” stress.

In Fig 5, we present Average Marginal Effects (AMEs) derived from the models reported in Table 3. In our application, the average marginal effects represent the effect of a one-unit change in the predictor on the probability of “increased” stress. Please note that “improved” serves as the reference category, so its average marginal effect is zero. The AMEs for electricity access and land amount show a similar pattern—the “remained the same” category is around 0.3 and the “decreased” category moves close to 0.10 (i.e., a 0.10 increase in the probability of “increased” stress). For water quality, “remained the same” has a small, possibly negative, effect, but the confidence interval indicates that it is not statistically significant. “Decreased” water quality, on the other hand, may heighten the probability of “increased” stress by some 0.7. Water access has negative AMEs (i.e., lower probability of “Increased” stress) but neither is statistically significant, while those that report that sewage access “remained the same” has a somewhat higher probability of increased stress. The AMEs imply that the effect of any one loss of service may not be especially powerful, but some households likely experienced multiple losses, which could converge to markedly increase the probability of increased stress.

## Discussion

In this paper, we have showed that, despite resettlement and compensation efforts, communities near hydropower projects in the Madeira river basin in Brazil experienced increased stress levels associated with deterioration in access to infrastructure, such as water and sewage. Our results show that most respondents reported that their stress level had increased because of the dam construction. Following the literature [14, 55], we suggest that large hydropower projects, such as the Jirau and Santo Antônio dams, make some kinds of promised infrastructure more difficult to access or more expensive, possibly because of the sudden influx to the region of workers needed to construct the dams, because resources might be re-directed towards dam construction, in addition to the inflation caused by this boom, but also because of the conditions of the resettled communities. A very common finding across the literature is that when land is given as compensation, it is rarely if ever of the same quality as what people had before and thus this injustice is a source of substantial stress to resettled people [76]. This is true in our study for those communities which received land as compensation (e.g., Riacho Azul, Nova Mutum). For that reason, often dam builders prefer to compensate with cash or houses. These losses could increase stress. Using interaction terms, we also asked how being compensated and resettled might reduce these impacts. Our results indicate that individuals who lost access to infrastructure—particularly reduced land and access to water—experienced increased stress and the compensation and resettled program did not reduce this effect. Strain on infrastructure, loss of public services, and a loss of resources is one mechanism by which hydropower projects create a reduction of well-being.

The prior literature has often studied communities that were directly involved in compensation programs and were included in formal efforts to address the problems caused by dam projects [e.g., 61]. Our study allowed us to go one step further, since we selected communities that are located both upstream and downstream from the dam and are geographically diffuse (see Fig 1). The prevalence of impacts and associated stress in ostensibly far-away communities, that dam builders claimed would not be impacted (and therefore are not included in the environmental and social impact assessments), indicates that hydropower projects have impacts that reverberate throughout a relatively large geographic region. This increased stress existed for years after the dams were constructed. To some extent, these results lend support to Scudder’s argument that hydropower impacts are so large that a generation may pass before a community recovers [21] and points to the need for long-term research.

Compensation programs have the potential to redress the losses experienced by communities that are impacted by hydropower projects. To date, the literature has mostly been critical of these programs, arguing that they are often implemented with little consultation with communities, are generally insufficient, and often exclude damages and communities that are nevertheless impacted [27, 28]. In all our models, our indicator for compensation and resettlement was not statistically significant and the effect was small in practical terms. That is, compensated individuals did not report lower stress across multiple models, suggesting that they experienced impacts the same as the uncompensated and were not made better off through compensation alone. Further, our interaction models imply that compensation did not blunt the effect of a loss of resources. For instance, we found that decreased access to electricity increased stress, and this effect was not weakened, reduced, or otherwise altered by compensation programs. Resettled people claim to have been surprised with the high energy bills in their new homes, compared to the bills they used to pay in the past. It is worth mentioning that other social services, conditions for production in the communities and the necessary logistics foreseen in the Basic Environmental Plan (Plano Básico Ambiental–PBA, in the Portuguese acronym) (Santo Antônio Energia 2022) were not implemented or were only partially provided.

Each of the communities in this study suffered a variety of broken promises that may explain why compensation alone did not mitigate the stress felt by the communities. Compensation practices are typically neither transparent nor sufficient [19], and resettled families are rarely given much choice in where they can resettle, and whether they can still live close to friends and family. Dam proponents often make promises to improve water quality and sewage systems, while also promising jobs and economic development, a dubious promise [17]. Future research is needed to determine how, or even if, compensation programs can effectively redress the damages caused by hydropower projects, including psychological and social outcomes that are often not considered.

Despite the efforts of activists, scientists, and other concerned groups, the Brazilian government has plans to continue to expand hydropower in coming years. Other nations, like China, will likely build additional hydropower capacity to satisfy the energy needs of their growing economies [77]. Moving forward, we argue that compensation practices and consultation with communities must be improved, and efforts must be made to ensure that communities near dam projects can access key infrastructure and services. Importantly, this loss of access has real implications—for example, losing access to electricity through exponential price increases lead to higher stress levels. Dam builders and associated governments could implement programs to protect community infrastructure, services and resources, in addition to compensating individual households with cash payments or assets such as housing. Compensation should also consider losses in relevant skills, knowledge, bonds of cooperation among neighbors and merchants, and other intangible losses that come from the values and bonds community membership had created, relations to their previous land use, and community social structure. This more holistic approach to compensation has the potential to reduce stress, and improve well-being more broadly.

## Supporting information

S1 Table Ordinal logistic regression models for increased stress in the Madeira river basin, Brazil. (DOCX)

S1 Dataset (XLSX)
