# Supplementary material for: Large hydropower projects increase stress despite compensation efforts: Evidence from the Brazilian Amazon
Source: PLoS One. 2023 Jul 14;18(7):e0284760. doi: 10.1371/journal.pone.0284760 (PMC10348581; doi:10.1371/journal.pone.0284760)
Supplement: S1 Table — (DOCX) [file pone.0284760.s001.docx]

| **S1 Table. Ordinal logistic regression models for increased stress in the Madeira river basin, Brazil.** | | | | | | | | | | |
| --- | --- | --- | --- | --- | --- | --- | --- | --- | --- | --- |
|  | Electricity Access | | Land Amount | | Water Quality | | Water Access | | Sewage Access | |
|  | Baseline | Interaction | Baseline | Interaction | Baseline | Interaction | Baseline | Interaction | Baseline | Interaction |
|  | b(se) | b(se) | b(se) | b(se) | b(se) | b(se) | b(se) | b(se) | b(se) | b(se) |
| **Electricity Access** (*ref. increased*) | | | | | | | | | | |
| Remained the Same | 0.389* | 0.358 |  |  |  |  |  |  |  |  |
|  | (0.19) | (0.21) |  |  |  |  |  |  |  |  |
| *resettled and compensated |  | 0.155 |  |  |  |  |  |  |  |  |
|  |  | (0.53) |  |  |  |  |  |  |  |  |
| Decreased | 0.631* | 0.576* |  |  |  |  |  |  |  |  |
|  | (0.25) | (0.27) |  |  |  |  |  |  |  |  |
| *resettled and compensated |  | 0.268 |  |  |  |  |  |  |  |  |
|  |  | (0.63) |  |  |  |  |  |  |  |  |
| **Land Amount** (*ref. increased*) | | | | | | | | | | |
| Remained the Same |  |  | 0.413 | 0.256 |  |  |  |  |  |  |
|  |  |  | (0.25) | (0.29) |  |  |  |  |  |  |
| *resettled and compensated |  |  |  | 0.633 |  |  |  |  |  |  |
|  |  |  |  | (0.68) |  |  |  |  |  |  |
| Decreased |  |  | 0.910*** | 0.699* |  |  |  |  |  |  |
|  |  |  | (0.27) | (0.31) |  |  |  |  |  |  |
| *resettled and compensated |  |  |  | 0.814 |  |  |  |  |  |  |
|  |  |  |  | (0.61) |  |  |  |  |  |  |
| **Water Quality (***ref. increased***)** | | | | | | | | | | |
| Remained the Same |  |  |  |  | 0.025 | -0.136 |  |  |  |  |
|  |  |  |  |  | (0.23) | (0.26) |  |  |  |  |
| *resettled and compensated |  |  |  |  |  | 0.889 |  |  |  |  |
|  |  |  |  |  |  | (0.60) |  |  |  |  |
| Decreased |  |  |  |  | 0.788** | 0.666* |  |  |  |  |
|  |  |  |  |  | (0.27) | (0.30) |  |  |  |  |
| *resettled and compensated |  |  |  |  |  | 0.583 |  |  |  |  |
|  |  |  |  |  |  | (0.64) |  |  |  |  |
| **Water Access** (*ref. improved*) | | | | | | | | |  |  |
| Remained the Same |  |  |  |  |  |  | 0.166 | 0.137 |  |  |
|  |  |  |  |  |  |  | (0.23) | (0.28) |  |  |
| *resettled and compensated |  |  |  |  |  |  |  | 0.034 |  |  |
|  |  |  |  |  |  |  |  | (0.52) |  |  |
| Decreased |  |  |  |  |  |  | 0.751* | 0.636 |  |  |
|  |  |  |  |  |  |  | (0.33) | (0.38) |  |  |
| *resettled and compensated |  |  |  |  |  |  |  | 0.589 |  |  |
|  |  |  |  |  |  |  |  | (0.83) |  |  |
| **Sewage Access** *(ref. improved)* | | | | | | | | | | |
| Remained the Same |  |  |  |  |  |  |  |  | 0.645* | 0.610 |
|  |  |  |  |  |  |  |  |  | (0.30) | (0.41) |
| *resettled and compensated |  |  |  |  |  |  |  |  |  | 0.014 |
|  |  |  |  |  |  |  |  |  |  | (0.59) |
| Decreased |  |  |  |  |  |  |  |  | 0.892 | 0.513 |
|  |  |  |  |  |  |  |  |  | (0.54) | (0.66) |
| *resettled and compensated |  |  |  |  |  |  |  |  |  | 1.432 |
|  |  |  |  |  |  |  |  |  |  | (1.33) |
| **Status** (*ref. not resettled and compensated*) | | | | | | | | | | |
| Resettled and compensated | -0.195 | -0.295 | -0.341 | -0.903 | -0.265 | -0.861 | -0.193 | -0.281 | -0.145 | -0.222 |
|  | (0.25) | (0.34) | (0.26) | (0.50) | (0.24) | (0.50) | (0.25) | (0.41) | (0.25) | (0.52) |
| **Sex** (*ref. female*) | | | | | | | | | | |
| Male | -0.120 | -0.126 | -0.120 | -0.135 | -0.127 | -0.117 | -0.175 | -0.182 | -0.201 | -0.188 |
|  | (0.21) | (0.21) | (0.22) | (0.22) | (0.21) | (0.21) | (0.21) | (0.21) | (0.21) | (0.21) |
| **Education** (*ref. no formal education*) | | | | | | | | | | |
| Primary Education | 0.389 | 0.383 | 0.489 | 0.472 | 0.420 | 0.367 | 0.452 | 0.466 | 0.453 | 0.459 |
|  | (0.25) | (0.25) | (0.26) | (0.26) | (0.25) | (0.25) | (0.25) | (0.25) | (0.25) | (0.25) |
| Secondary | 0.612* | 0.602* | 0.838** | 0.827** | 0.566* | 0.525 | 0.619* | 0.623* | 0.668* | 0.671* |
|  | (0.28) | (0.28) | (0.30) | (0.30) | (0.28) | (0.28) | (0.28) | (0.28) | (0.28) | (0.28) |
| Post-Secondary/ Technical | 0.459 | 0.454 | 0.804* | 0.760 | 0.459 | 0.441 | 0.480 | 0.497 | 0.519 | 0.527 |
|  | (0.36) | (0.36) | (0.39) | (0.39) | (0.36) | (0.36) | (0.36) | (0.36) | (0.36) | (0.36) |
| **Sex of the head of household** (*ref. male*) | | | | | | | | | | |
| Female Head of Household | 0.094 | 0.085 | 0.067 | 0.047 | 0.082 | 0.102 | 0.041 | 0.045 | 0.031 | 0.055 |
|  | (0.23) | (0.23) | (0.25) | (0.25) | (0.23) | (0.23) | (0.23) | (0.23) | (0.23) | (0.23) |
| **Community type** (*ref. downstream*) | | | | | | | | | | |
| Upstream | 0.387* | 0.389* | 0.506** | 0.494* | 0.205 | 0.189 | 0.339 | 0.342 | 0.475** | 0.474** |
|  | (0.18) | (0.18) | (0.19) | (0.19) | (0.18) | (0.18) | (0.18) | (0.18) | (0.18) | (0.18) |
| AIC | 1116.953 | 1120.743 | 987.543 | 989.728 | 1121.049 | 1122.840 | 1126.810 | 1130.231 | 1131.653 | 1134.156 |
| BIC | 1165.960 | 1178.661 | 1035.593 | 1046.514 | 1170.228 | 1180.961 | 1175.938 | 1188.291 | 1180.832 | 1192.276 |

Note: N=554. *** for p<0.001, ** for p<0.01 and * for p <0.05. Data was gathered from August 2019 to March 2020.
